# On the Use of Formaldehyde in Atrophic Rhinitis

**Published:** 1898-05

**Authors:** Geo. L. Richards

**Affiliations:** Fall River, Mass.; Otologist and Laryngologist to the Fall River and Emergency Hospitals


### ON THE USE OF FORMALDEHYDE IN ATROPHIC RHINITIS.

By GEO. L. RICHARDS, M.D., Fall River, Mass.

Otologist and Laryngologist to the Fall River and Emergency Hospitals.

I offer the followlng brief note as a contribution to the therapeutics of atrophic rhinitis, believing that anything in the way of an addition to our armamentarium in this disease will be welcomed. Remedies innumerable have been and are being tried with more or less success. There is no specific, and, up to the present time, the treatment can be formulated in a few words. Cleanliness and stimulant applications, to promote a freer and thinner secretion, has been about all that we have been able to accomplish.

Observing the powerful germicidal properties of aqueous solutions of formaldehyde, it occurred to me to try what effect it would have when used in atrophic rhinitis; not to displace any treatment which might be in use, but as an adjunct, for I have not myself used it alone to the exclusion of other remedies. I have used it as follows: After removal of all the crusts and debris with a weak alkaline solution, by means of a syringe and cotton applicators, I have then washed out each nostril thoroughly with a solution of formaldehyde, containing about five to ten drops of the forty per cent. solution to eight ounces of warm water. As it is very irritating, even in dilute solutions, a preliminary spraying of the nose with cocaine is advisable. It produces always a sense of smarting throughout all of the nasal mucous membrane with which it comes in contact, lasting, however, but a short time. At home I have one drop added to the solution which the patient uses in the douche cup for the daily cleansing. Under its use the crusts diminish in number and all unpleasant odor ceases. This is reported as a preliminary note, with the hope that others will try the remedy and report on the same.—*The Laryngoscope.*

